# Association of Viral Infections With Oral Cavity Lesions: Role of SARS-CoV-2 Infection

**DOI:** 10.3389/fmed.2020.571214

**Published:** 2021-01-14

**Authors:** Giusy Rita Maria La Rosa, Massimo Libra, Rocco De Pasquale, Sebastiano Ferlito, Eugenio Pedullà

**Affiliations:** ^1^Department of General Surgery and Medical-Surgical Specialties, University of Catania, Catania, Italy; ^2^Department of Biomedical and Biotechnological Sciences, Oncologic, Clinic and General Pathology Section, University of Catania, Catania, Italy

**Keywords:** COVID-19, novel coronavirus, oral health, oral lesions, oral mucosa, SARS-CoV-2, viral infection

## Abstract

Different viral agents, such as herpesviruses, human papillomavirus, and Coxsackie virus, are responsible for primary oral lesions, while other viruses, such as human immunodeficiency virus, affect the oral cavity due to immune system weakness. Interestingly, it has been reported that coronavirus disease 2019 (COVID-19) patients can show cutaneous manifestations, including the oral cavity. However, the association between oral injuries and severe acute respiratory syndrome coronavirus 2 (SARS-CoV-2) infection is still unclear. This narrative review aimed to summarize the available literature and provide an overview of oral lesions associated with COVID-19. An online literature search was conducted to select relevant studies published up to November 2020. The results of 17 studies showed variability in oral lesions associated with COVID-19, including ulcerations, aphthous-like lesions, and macules. The tongue, lips, and palate were the most frequent anatomical locations. According to current knowledge, the etiopathogenesis of multiple COVID-19-associated lesions seems to be multifactorial. The appearance of such lesions could be related to the direct or indirect action of SARS-CoV-2 over the oral mucosa cells, coinfections, immunity impairment, and adverse drug reactions. Nevertheless, COVID-19-associated oral lesions may be underreported, mainly due to lockdown periods and the lack of mandatory dispositive protection. Consequently, further research is necessary to determine the diagnostic and pathological significance of oral manifestations of COVID-19. All medical doctors, dentists, and dermatologists are encouraged to perform an accurate and thorough oral examination of all suspected and confirmed COVID-19 cases to recognize the disease's possible early manifestations.

## Introduction

The oral cavity is particularly susceptible to viral infections because of its conformation, particularly its soft tissue and salivary glands. Several viruses, including herpes simplex virus (HSV) and human papillomavirus (HPV), are associated with oral disease-causing primary lesions. Furthermore, oral mucosa can be affected by the secondary pathological processes of a bacterial or fungal nature due to viral immunosuppression, such as the human immunodeficiency virus (HIV). Consequently, the oral cavity could be considered a “biological barometer” of the viral immunosuppression advancement. Moreover, an implication of certain viral agents has been seen in dysplastic and neoplastic transformations of squamous epithelium (i.e., HPV) (1, 2). General and specialist dentists play a crucial role in evaluating, diagnosing, and managing such lesions, particularly considering the impacts of oral diseases on overall health and quality of life.

A new coronavirus, known as severe acute respiratory syndrome coronavirus 2 (SARS-CoV-2), responsible for coronavirus disease 2019 (COVID-19), was identified in Wuhan, Hubei Province, China, at the end of 2019. It then spread worldwide, becoming a global pandemic (3).

The main transmission route is via large respiratory droplets, even though the virus has also been identified in the stool and urine of affected individuals. It presents great variability in the severity of clinical manifestations, such as dry cough, shortness of breath, and fever (4, 5), passing from a mild flu-like illness to severe respiratory syndrome. Mortality rates vary according to region and change as the number of affected individuals is updated (6). Angiotensin-converting enzyme 2 (ACE2) receptor is considered the main functional receptor through which SARS-CoV-2 infects cells. The wide expression of ACE2 receptors in different anatomical sites, including the respiratory and gastrointestinal tracts, could explain the variability of reported clinical manifestations (7).

Interestingly, dermatological manifestations have also been observed in some patients affected by COVID-19. The most common skin lesions in these subjects include erythematous rash, urticaria, and vesicle formation, especially localized on the trunk, which seems to be the anatomic region most involved (5, 8). Oral lesions, such as unspecific ulceration, desquamative gingivitis, petechiae, and coinfections, such as candidiasis (9–13), have also been reported. Moreover, Xu et al. (14) reported a high level of expression of ACE2 receptors in epithelial cells of the oral mucosa, particularly tongue epithelial cells. These results suggest that oral mucosa could be a target of SARS-CoV-2 infection. Nevertheless, it is still unclear whether these manifestations are a specific clinical pattern derived from direct SARS-CoV-2 infection or a consequence of systemic involvement because of the possibility of coinfections, a compromised immune system, and adverse reactions to medical treatment (13, 15, 16). Because the prevalence of clinical oral manifestations is still unknown, the range of COVID-19 manifestations in the oral mucosa is of broad and current interest.

Therefore, after a brief excursus into the main viral agents associated with oral mucosa lesions, this review aims to summarize the updated literature on oral lesions in patients with COVID-19 and emphasize their clinical implications.

### The Host Defense of Mouth and Viral Diseases

**Figure 1:**
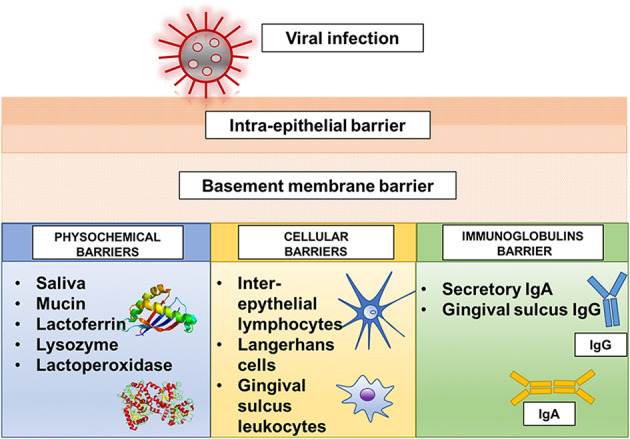
The principal host defense mechanisms in the oral cavity.

**Figure 2:**
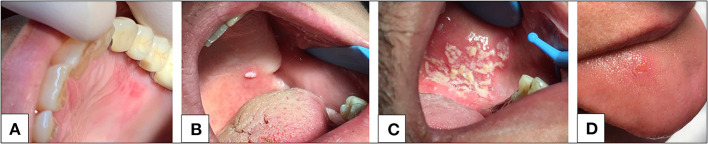
**(A)** Herpetic-like oral lesion. **(B)** Oral mucosal verruca lesion. **(C)** Opportunistic infection (candidiasis) in patient affected by HIV. **(D)** Unspecific ulcerous lesion in a SARS-CoV-2 infected patient. Image 2D is a case courtesy of Chaux-Bodard et al. (9).

The oral cavity possesses a series of physicochemical, cellular, and immunoglobulin barriers that prevent the entrance of harmful substances and microorganisms (2) (Figure 1). However, physicochemical barriers within the oral mucosa, including saliva and oral epithelium, are not absolute. The saliva secreted by the major and minor salivary glands contains many non-specifically protective agents, such as mucin, lysozyme, lactoperoxidase, and lactoferrin. In particular, lactoferrin, an iron-binding glycoprotein of the transferrin family, can inactivate many deoxyribonucleic acid (DNA) and ribonucleic acid (RNA) viruses, including cytomegalovirus, HSV, and rotavirus (17, 18). Cellular barriers involve the cells of the gingival sulcus, inter-epithelial lymphocytes, and Langerhans cells. In particular, Langerhans cells, which are dendritic inter-epithelial cells and act as mucosa “sentinels,” are localized in the mouth inverse to the degree of oral mucosa keratinization and are primarily implicated in immune reactions (2, 19, 20). Despite these defense mechanisms, the oral mucosa is particularly subjected to viral infections (Figure 2). A virus is a sub-microscopic entity formed by a protein shell (known as a capsid) surrounding a single nucleic acid, DNA or RNA, only able to replicate in bacterial, animal, and vegetal cells (21). Viral genetic material is distinguishable from human genetic material due to its unique chemical and/or physical features. Also, a lipid envelope derived from the host cell membrane can be identified in some viruses (22, 23). Even though viral infection can involve any human cell, the oral cavity offers an ideal entry into a new host (24). Some of the most well-known viral agents associated with oral lesions are HSVs and HPVs. HSVs contain a double-stranded linear DNA molecule enveloped by an icosahedral capsid and a lipid casing (25). Initially involved in primary infections, they then remain dormant but can later cause secondary or recurrent infections. Eight types of HSV have been identified as human pathogens, and most are responsible for oral diseases (1, 23, 25–35). HPVs are non-enveloped viruses containing double-stranded DNA (36). Over 100 subtypes of HPV have been identified, with at least 13 correlated to an insurgence of oral lesions (37–42). The oral wart is a generic term used to identify all papillary and verrucal proliferations. Squamous papilloma is one of the most represented papillary lesions in the oral cavity (1, 36). Oral lesions can also be secondary due to an immunosuppression state, such as occurs in HIV infection (43, 44). HIV is part of the *Lentivirus* genus, part of the *Orthoretroviridae* subfamily of the *Retroviridae* family. Two identical single-stranded RNA molecules form the HIV genome. The most frequent oral manifestations of HIV infection are opportunistic infections, such as candidiasis (45, 46), and malignancies, such as Kaposi's sarcoma (47, 48). The other most important viral agents associated with oral lesions are reported in Table 1. The association between oral lesions and SARS-CoV-2 remains controversial.

**Table 1:** Viruses associated with oral lesions.

**Viral family**	**Virus**	**Oral disease**	**Oral lesion**
Herpesvirus (HSV)	HSV-1 HSV-2 HSV-3 (Varicella zoster) HSV-4 (Epstein Barr) HSV-5 (Citomegalovirus) HSV-6 HSV-7 HSV-8	Primary herpetic gingivostomatitis Herpes labialis (recurrent infection) Similar to HSV-1 but rare Primary infection (rare) Recurrent infection Mononucleosis Burkitt's lymphoma Nasopharyngeal carcinoma Sialadenopathy Aphthae - - Kaposi's sarcoma	Vesicles Erosions Ulcers
Papillomavirus (HPV)	More than 100 subtypes: HPV-2,−6,−11,−57 HPV-6 and−11 HPV-13 and−32 HPV-16 and−18	Squamous papillomas Verruca vulgaris Condyloma acuminatum Focal epithelial hyperplasia (Heck's disease) Dysplastic and neoplastic transformations of squamous epithelium	Exophytic papillary lesions Multiple, pink, soft tissue masses
Poxvirus	Variola Molluscum contagiosum (MCV)	Smallpox Molluscum contagiosum	Maculopapular lesions Erythematous papules
Picornavirus	Coxsackie virus: A16, A6 A1-A6, A8, A10, A22	Hand, foot, and mouth disease Herpangina	Vesicles Ulcers
Paramyxovirus	Measles virus (MV) Mumps virus	Rubeola Mumps	Small erythematous macules with white necrotic center (Koplik's spots) Ulcers
Retrovirus	Human immunodeficiency virus (HIV)	Opportunistic infections (viral, bacterial, fungal) Malignancies	Not typical lesions but dependent on the secondary lesion

## Materials and Methods

An electronic search was conducted in PubMed, Scopus, and Web of Science for literature updated to November 20, 2020. A combination of the following keywords was used: “oral mucosal lesions” OR “oral lesions” AND “COVID-19” OR “SARS-CoV-2” OR “novel coronavirus disease.” The full-text articles of all potential studies were evaluated, and the references cited by the relevant studies were manually searched for further studies. Given the lack of available data, all types of studies reporting oral mucosal lesions in patients with laboratory-confirmed COVID-19 were included; only literature reviews were excluded. However, we decided to include only those with laboratory-confirmed COVID-19 when evaluating the reported cases; suspected COVID-19 were excluded. Other exclusion criteria were articles for which the full text was not accessible or not available in English. Duplicate articles were removed, and a first screening was performed by reading only the titles and abstracts of the studies.

## Results

From the 86 studies retrieved, only 17 satisfied the inclusion criteria, of which 11 were letters to the editor, 3 were case reports, 2 were case series, and 1 was a short communication. Table 2 provides a detailed description of the cases included. Given that the studies were published between April and November 2020, the results were listed in the table in alphabetical order of the first author's surname. Excluding the only study in which the gender and age of the participants were not reported (58), 33 cases were female and 24 cases were male. The mean age of reported cases was 42.92 ± 18.05.

**Table 2:** General aspects of the included studies.

**Study**	**Design**	**Sample (n)**	**Age and gender**	**Oral lesion**	**Localization**	**Time on onset (days)^a^**
Amorim Dos Santos et al. (62)	Case report	1	67 (M)	White plaque; pinpoint yellowish ulcers	Tongue dorsum	24
Ansari et al. (49)	Letter to the Editor	2	75 (M) and 56 (F)	Ulcer	Hard palate and tongue	6 ± 1.41
Bezerra et al. (50)	Letter to the Editor	1	33 (M)	Ulceration; ulcer with necrotic background	Floor of mouth; retromolar region and lip mucosa	70 (first appearance) and 90 (second)
Brandão et al. (51)	Case series	8	53.87 ± 24.86; 5 (M) and 3 (F)	Aphthous-like + necrosis; hemorrhagic ulceration with necrotic areas; aphthous-like; petechia	Upper and lower lip mucosa; anterior dorsal tongue; lateral borders of the tongue; ventral portion of tongue; upper and lower labial mucosa; tonsillar pilar	6 ± 2.56
Carreras-Presas et al. (10)	Short communication	1	65 (F)	Blisters; desquamative gingivitis	Internal lip mucosa	22
Cebeci Kahraman et al. (52)	Letter to the Editor	1	51 (M)	Erythema; petechia; pustular enanthema	Hard palate; soft palate border	10
Chaux-Bodard et al. (9)	Letter to the Editor	1	45 (F)	Erythematous macula evolved into ulcer	Tongue	8 days before the laboratory-confirmed COVID-19
Ciccarese et al. (53)	Letter to the Editor	1	19 (F)	Erosion; ulceration and petechia	Palate and lips	7
Corchuelo and Ulloa (63)	Case report	1	40 (F)	Reddish plaque; dark brown hyperpigmentation	Lower lip; gum	21
Cruz Tapia et al. (54)	Case series	4	47 ± 7.11; 1 (M) and 3 (F)	Bulla; macula; papule-plaque	Hard palate; tongue	N.A.
Díaz Rodríguez et al. (55)	Letter to the Editor	3	58 ± 18.02; 1 (M) and 2 (F)	Aphthous-like; tongue depapillation; fissures; red plate	Dorsum of the tongue; labial commissure; palate	N.A.
Dominguez-Santas et al. (56)	Letter to the Editor	4	33 ± 10.19; 3 (M) and 1 (F)	Minor aphthae	Buccal and labial mucosa; tongue	3 ± 2.16
Kitakawa et al. (57)	Case report	1	20 (F)	Vesicle	Median lower lip semimucosa	7
Nuno-Gonzalez et al. (58)	Research letter	78	N.A.	Lingual papillitis; glossitis; aphthous-like	Tongue; oral mucosa	N.A.
Riad et al. (61)	Letter to the Editor	26	36.81 ± 15.65; 9 (M) and 17 (F)	Ulcer	Tongue	4.12 ± 1.39
Sakaida et al. (59)	Letter to the Editor	1	52 (F)	Erosion	Lips; buccal mucosa	N.A.
Soares et al. (60)	Letter to the Editor	1	42 (M)	Ulceration and reddish macula	Hard palate, tongue and lips	N.A.

*n = 17. M, Male; F, Female; N.A., Not available*.

a *The time on onset refers to days passed after the laboratory-confirmed diagnosis of COVID-19*.

The documented manifestations of oral mucosa were quite heterogeneous, varying in the kind of lesion and the location. The most frequent findings were ulcerations (9, 50, 60–62), sometimes associated with necrotic areas (50, 51), aphthous-like lesions (51, 55, 56, 58), and petechiae (51, 52, 54). Maculae (53, 60), blisters (10, 57), lingual papillitis or depapillation (58), and erythema or red plaques (52, 63) were also among the described oral lesions. Besides, a case of dark brown hyperpigmentation was documented by Corchuelo and Ulloa (63).

Tongue (9, 51, 54, 55, 58–62), lips (10, 50, 51, 57, 59, 60, 63) and palate (52, 54, 60) were the most frequently described anatomical locations.

## Discussion

An increasing number of atypical clinical presentations have been reported during SARS-CoV-2 infection, including dermatological and oral manifestations (16, 64–67). The pathogenesis of skin damage during COVID-19 is not well known, but some hypotheses have been formulated. For example, the presence of viral particles in cutaneous blood vessels could induce lymphocytic vasculitis through cytokine production, i.e., interleukin-1 (IL-1), interferon gamma (IFN-γ), and tumor necrosis factor alfa (TNF-α) by CD4+ T helper lymphocytes and the migration of eosinophils, CD8+ cytotoxic T cells, B cells, and natural killer (NK) cells (68, 69). Another possible explanation of cutaneous disturbances correlated to SARS-CoV-2 is the formation and accumulation of microthromboses, which could reduce the blood flow to the cutaneous microvasculature (70), and the presence of deoxygenated blood in venous plexi could further contribute to these cutaneous lesions. Moreover, the deposition of complement components C5b-9 and C4d in pauci-inflammatory thrombogenic vasculopathy and their co-localization with COVID-19 spike glycoproteins were shown by Magro et al. (71). It is reasonable to hypothesize that skin involvement is due to a combination of these mechanisms rather than a single one (5). Taste disorders were the most common oral symptom in patients with COVID-19, probably due to a local inflammatory response resulting from rhinitis triggers, which can hamper taste buds' normal function (15, 72). Additionally, oral mucosa involvement was described during SARS-CoV-2 infection. Since the first description of oral lesions in SARS-CoV-2 positive patients, reported by Martín Carreras-Presas et al. (10), several more recent studies have also reported oral mucosa lesions in COVID-19, such as ulcers (50, 60–62), aphthae (51, 55), and maculae (53, 60). The clinical significance of oral mucosa involvement during SARS-CoV-2 infection remains controversial.

As previously reported (14), the high expression of ACE2 on oral epithelial cells, especially on the tongue, suggests that the oral cavity might be an anatomical site particularly susceptible to SARS-CoV-2 infection. Consequently, as suggested by Brandão et al. (51), the interaction between SARS-CoV-2 and ACE2 might disrupt the oral keratinocytes' function, resulting in painful oral ulcers. Furthermore, oral mucosa lesions during COVID-19 could be justified by the variable inflammatory reaction, which can induce vascular inflammation, as observed for cutaneous manifestations (5, 73). The most recent publications on oral mucosa lesions in patients affected by COVID-19 support an association with organic damage and/or complications for thrombocytopenia, anticoagulant therapy, disseminated intravascular coagulation, and systemic inflammation (60, 62, 63). According to Cruz Tapia et al. (54), clinical manifestations and histological findings suggest the possibility that the oral cavity presents the primary or secondary alterations of vascular-hematologic damage associated with COVID-19. Nevertheless, as reported by Martín Carreras-Presas et al. (10) and Hedou et al. (74), ulcers or vesiculobullous lesions can occur as in other viral infections. It is largely documented that high levels of fatigue and stress can increase the risk of the reactivation of HSV (75).

Moreover, oral damage could also be a manifestation of an immunosuppression state and microbiome dysbiosis caused by a viral infection (76). According to Bezerra et al. (50), it is reasonable to think that COVID-19 systemic immune deregulation may cause a more prolonged immune imbalance, which could predispose these late, secondary oral lesions. In addition, as stated by de Sousa et al. (13), most patients developed oral mucosal injury during the hospitalization period, which supports the hypothesis of coinfections, immunity impairment, and adverse reactions to COVID-19 treatment medications.

Interestingly, Martín Carreras-Presas et al. (10) suggested that oral lesions, such as ulcers, could be an inaugural symptom of COVID-19. According to Amorim Dos Santos et al. (15), in mild cases, oral mucosal lesions occurred before or at the same time as the initial respiratory symptoms; however, in those who required medication and hospitalization, the lesions developed approximately 7–24 days after the onset of symptoms. Limited to the cases reported, the time to onset was variable, ranging from 4 to 90 days; however, the time on onset was unavailable in several reported studies, confirming the necessity to verify this data in larger patients' cohorts.

Regarding the age of patients presenting with oral injuries, Brandão et al. (51) reported two distinct oral lesions patterns. One was represented by aphthous-like ulcers in young patients with mild cases of COVID-19, and the other resembled HSV-1 necrotic ulcers in more severe cases of immunosuppressed older patients. Again, the lack of data available from a large sample of participants makes further investigations necessary to support these hypotheses.

Another interesting fact emerging from the reported cases is how online professional consultation using photography (telemedicine) could be a very useful, additional tool to support clinicians in the early diagnosis of oral lesions, especially when direct observation is not possible (57, 63). Indeed, an early clinical diagnosis and the development of sensitive diagnostic tools are essential for a correct management of the disease (77, 78).

There are some limitations to underline in this study. First, almost all the reports were published as letters to the editor, thus imposing editorial limitations that reduce reporting comprehensibility (12). Moreover, the oral manifestations' real incidence could have been underestimated due to exposure and contamination risk while conducting photographic imaging (15) and the limited data available on oral lesion injuries in asymptomatic patients. Another important aspect to consider is the limited availability of microscopic and histological data of oral mucosa lesions in COVID-19. The only accessible data referred to histological characterizations performed by Soares et al. (60) and Ansari et al. (49), which confirmed the presence of an inflammatory infiltrate, suggesting that the patients' lesions could be associated with COVID-19 disease. It is auspicious that the characterization of the oral lesions of COVID-19 infected patients should include incisional biopsies, followed by direct viral testing for SARS-CoV-2, as suggested by Brandão et al. (51).

Further research is necessary to determine the diagnostic and pathological significance of oral manifestations during COVID-19. Indeed, oral mucosa involvement during viral infection may assume differing clinical significance: it could represent either the first sign of viral disease or coexist as a co-symptom or represent a unique sign of the viral infection (26). In this context, the importance of the clinical oral examination of patients with confirmed or suspicious COVID-19 infection should be emphasized, given the need for support, pain control, and quality of life. In addition, dental operators are potentially exposed to a high degree of contamination with SARS-CoV-2 because of dental procedures that produce aerosols (79, 80). Consequently, an accurate inspection of the oral cavity, always with the mandatory dispositive protection (79), could be crucial in the dental setting to perform a more accurate triage of patients and improve safety operator, avoiding underestimation and misdiagnoses of oral signs and symptoms.

## Conclusions

The new SARS-CoV-2 responsible for the global COVID-19 pandemic has become a sanitary emergency of primary importance. Although the typical symptoms include fever, shortness of breath, and a dry cough, cutaneous manifestations have also been reported, including some oral lesions. An association between oral diseases and SARS-CoV-2 infection is still unclear and currently poorly investigated. The appearance of such lesions could be related to the direct or indirect action of SARS-CoV-2 over the oral mucosa cells, coinfections, immunity impairment, and adverse drug reactions. Nevertheless, oral manifestations of this disease seem to be underreported, especially due to lockdown periods and the lack of mandatory dispositive protection. Consequently, based on these outcomes, we can conclude that (1) further studies are necessary to establish the diagnostic and pathological significance of oral manifestations during COVID-19; (2) the oral examination in patients with COVID-19 should not be unattended but rather promote a specialist multidisciplinary approach, including especially dental practitioners; (3) early recognition of oral lesions associated to COVID-19 could be crucial in the dental setting to perform a more accurate triage of patients and improve operator safety, avoiding underestimation and misdiagnosis of oral manifestations.

## Author Contributions

GL, ML, and EP contributed to all steps of manuscript preparation. RD and SF participated in editing and critical revision of article. All authors contributed to the article and approved the submitted version.

## Conflict of Interest

The authors declare that the research was conducted in the absence of any commercial or financial relationships that could be construed as a potential conflict of interest.
